# Summary and review of data on psychiatric emergency care in 2023 in a hospital in the Community of Madrid

**DOI:** 10.1192/j.eurpsy.2025.1454

**Published:** 2025-08-26

**Authors:** L. Fontecha, R. Fernández

**Affiliations:** 1 Hospital Universitario Infanta Cristina, Parla, Spain

## Abstract

**Introduction:**

The term ‘*psychiatric emergency*’ refers to any situation in which certain psychopathological symptoms or behavioural disorders are perceived as disturbing, worrying or threatening by the patient, their family/social environment or other health/social actors, thus motivating the request for urgent care.

**Objectives:**

The aim of this poster is to analyse the data on psychiatric emergency care in 2023 at the Infanta Cristina University Hospital in Parla.

**Methods:**

The computer record of all psychiatric care carried out in the hospital’s Emergency Department during 2023 has been reviewed.

**Results:**

During 2023, a total of 1231 psychiatric emergencies were registered, of which 136 (11.05%) belonged to the child and adolescent population and 1095 (88.05%) to the adult population (i.e., over 18 years of age).

Of the 136 attendances in the child and adolescent population, 93 (68.38%) were of people identified as female and 43 (31.62%) as male.

Of the 1095 attendances in the adult population, 641 (58.54%) were of people identified as female and 444 (40.55%) as male.

In both the child and adolescent population and the adult population, the most frequent reason for consultation was “anxiety” (25% and 32.05%, respectively). However, it should be noted that, in both populations, two of the most frequent reasons for consultation were “autolytic ideation” (19.12% and 16.25%, respectively) and “autolytic attempt” (19.85% and 12.05%, respectively).

**Conclusions:**

Women went to the psychiatric emergency department more frequently.

The percentage of children and adolescents who attended the psychiatric emergency department was high.

Although the most frequent reason for consultation was “anxiety”, it is important to be able to reflect on the high figures of “autolytic ideation” and “autolytic attempt”.

**Disclosure of Interest:**

None Declared

